# Variations in greenhouse gas emissions of individual diets: Associations between the greenhouse gas emissions and nutrient intake in the United Kingdom

**DOI:** 10.1371/journal.pone.0259418

**Published:** 2021-11-23

**Authors:** Holly L. Rippin, Janet E. Cade, Lea Berrang-Ford, Tim G. Benton, Neil Hancock, Darren C. Greenwood

**Affiliations:** 1 School of Medicine, University of Leeds, Leeds, United Kingdom; 2 School of Food Science and Nutrition, University of Leeds, Leeds, United Kingdom; 3 Priestley International Centre for Climate, University of Leeds, Leeds, United Kingdom; 4 School of Biology, University of Leeds, Leeds, United Kingdom; 5 Royal Institute of International Affairs, Chatham House, London, United Kingdom; 6 Leeds Institute for Data Analytics, University of Leeds, Leeds, United Kingdom; University of Rhode Island, UNITED STATES

## Abstract

**Background:**

Food production accounts for 30% of global greenhouse gas (GHG) emissions. Less environmentally sustainable diets are also often more processed, energy-dense and nutrient-poor. To date, the environmental impact of diets have mostly been based on a limited number of broad food groups.

**Objectives:**

We link GHG emissions to over 3000 foods, assessing associations between individuals’ GHG emissions, their nutrient requirements and their demographic characteristics. We also identify additional information required in dietary assessment to generate more accurate environmental impact data for individual-level diets.

**Methods:**

GHG emissions of individual foods, including process stages prior to retail, were added to the UK Composition Of Foods Integrated Dataset (COFID) composition tables and linked to automated online dietary assessment for 212 adults over three 24-hour periods. Variations in GHG emissions were explored by dietary pattern, demographic characteristics and World Health Organization Recommended Nutrient Intakes (RNIs).

**Results:**

GHG emissions estimates were linked to 98% (n = 3233) of food items. Meat explained 32% of diet-related GHG emissions; 15% from drinks; 14% from dairy; and 8% from cakes, biscuits and confectionery. Non-vegetarian diets had GHG emissions 59% (95% CI 18%, 115%) higher than vegetarian. Men had 41% (20%, 64%) higher GHG emissions than women. Individuals meeting RNIs for saturated fats, carbohydrates and sodium had lower GHG emissions compared to those exceeding the RNI.

**Discussion:**

Policies encouraging sustainable diets should focus on plant-based diets. Substituting tea, coffee and alcohol with more sustainable alternatives, whilst reducing less nutritious sweet snacks, presents further opportunities. Healthier diets had lower GHG emissions, demonstrating consistency between planetary and personal health. Further detail could be gained from incorporating brand, production methods, post-retail emissions, country of origin, and additional environmental impact indicators.

## Introduction

Providing populations with healthy, nutritionally adequate diets that are environmentally sustainable is a global challenge, with food production accounting for 30% of greenhouse gas (GHG) emissions [1]. As production efficiency has increased, food-system efficiency in sustainably providing nutritious food has declined, and the cost of dietary ill-health and environmental degradation now exceeds the economic value of agriculture [2]. The societal-level impact of diet is addressed by the EAT Lancet Commission [3], targeting food production systems that deliver diets to promote both human and planetary health. The environmental costs of our diets include impacts on air and water quality, water availability, soil health, biodiversity loss and homogenization of landscapes. They also contribute to poor human health through exposure to poor air quality, antimicrobial resistance from antibiotic use and poor dietary health associated with noncommunicable diseases [3].

At an individual-level, personal health is partly reliant on planetary health and vice versa, through making low impact lifestyle choices. Unhealthy, environmentally unsustainable diets are often processed, energy-dense and nutrient-poor, for example high in saturated fat. A healthy diet based on vegetables, fruit, wholegrains, legumes, nuts and unsaturated oils, with small amounts of seafood and poultry, is also a sustainable one [3]. This implies diets should contain minimal red meat, processed meat, with less added sugar or refined grains [3]. Wider use of this type of diet could prevent a fifth of premature adult deaths whilst reducing GHG emissions by four fifths [1]. More sustainable diets have also been shown to have better diet quality scores based on a range of indicators [4, 5]. However, research supporting this is based on a limited number of food items or food groups. This can lead to conflicting advice, such as sugar-sweetened beverages having low environmental impact [6, 7].

Dietary assessment tools, which estimate nutrient intake derived from the composition of thousands of food items, rarely estimate their environmental impact. Instead, measures of environmental sustainability are often derived broader food groups. To move beyond general advice at the population level, to specific advice tailored to the individual, requires measures of environmental sustainability applied to a comprehensive range of specific food items at a more granular level.

We therefore aimed to identify and link GHG emissions values across the food items covered by the UK Composition Of Foods Integrated Dataset (COFID) [8], generating a GHG emissions estimate for each individual diet in a cohort of 212 adults [9, 10]. We evaluate the associations between the GHG emissions, the nutrient requirements, and the demographic characteristics of the participants. Based on this, we aim to identify the additional information required in dietary assessment methods to generate more accurate indicators of environmental impact for individual-level diets. For this initial proof of concept, we chose to report on GHG emissions, rather than land and water use, or acidifying and eutrophying emissions, as this is where associations between health and environmental gains have previously appeared strongest [3].

## Methods

### Update of the UK COFID database

#### Identification of greenhouse gas emissions from the literature

Relevant databases and papers (both LCA studies and meta-analyses) including details of the GHG emissions values of foods were systematically identified, starting with three recent systematic reviews [6, 11, 12] using cited original research and earlier reviews. These, in turn, were searched for further relevant sources, until no more were found. Supplementary materials and appendices were reviewed, containing relevant information on GHG emissions, life cycle analysis (LCA) studies and conversion factors. Key authors were contacted to request additional information and underlying databases.

Seven references were identified by the authors as key references containing details of GHG emissions for a range of foods or food groups, as opposed to a single LCA study or group of LCAs for a single food. S1 Appendix summarises relevant information for each key reference, including the number of foods for which GHG emissions values were given, the unit of measure, method of deriving the GHG emissions values, the process boundary and any gaps in the foods covered. The food-specific LCA studies and LCA meta-analyses used to generate GHG emissions values for the food items included in each key reference were tabled (see S2 Appendix). Using this list and the information tabled in S1 Appendix, the most comprehensive, consistent, and compatible of the seven key references were identified and the documented GHG emissions values were used as the basis of those assigned to generic food items in the myfood24 dietary database. The key references chosen were Poore & Nemecek [12], Murakami & Livingstone [4] (n = 1244) and Green et al. [6].

The author of the key reference from which most food GHG emissions values were taken [12] was contacted and shared details of the underlying tool used to generate GHG emissions and other environmental indicator values. This algorithm was set up at a detailed food item level and accounted for factors including production method, land use management, feed used, soil and climate, processing and transport of both the product and aspects of its production e.g., fertiliser and feed. It enabled the assignation of GHG emissions values for a greater number of food items on a more detailed level, for example, separate values for almonds, peanuts and hazelnuts rather than a single value for all nuts. It also accounted for LCA origin; for example, where LCAs were not specific to foods from the UK, the impact on GHG emissions relative to the UK food system was built into the algorithm. This meant that the algorithm presented estimates that were representative of food consumed in the UK. Values taken from the other two references [6, 16] were based on foods consumed in the UK.

The GHG emissions values assigned to the generic food items included process stages up to the point of retail [6]. Some sources of GHG emissions values were used that included the whole life cycle up to the point of consumption. These were converted to the point of retail using conversion factors specified in the literature: GHG emissions values were multiplied by 0.8 to account for the 20% contribution of post-retail life cycle stages in food items requiring refrigeration. GHG emissions values for food items not requiring refrigeration were multiplied by 0·85 [6].

#### Data linkage

GHG emissions values for foods in the identified literature sources were matched to the corresponding generic food items from the UK COFID version 7 [8]. This was carried out in the database associated with the dietary assessment tool myfood24. The myfood24 tool was developed as an online self-administered 24hr dietary recall, underpinned by a large database using the COFID data of over 3000 generic foods which has been linked to 40,000 branded items [9, 10].

Multiple generic food items were assigned common GHG emissions values depending on the closest match according to food type, nutritional value, and climate impact. Similarly, proxy values were used when no GHG emissions value existed for a generic food item in the myfood24 database. For example, buckwheat and millet were assigned the GHG emissions value for ‘other grains’. Human milk was assumed to have zero climate impact and given a GHG emissions value of 0. Water was assumed to be tap water, unless one of the brands of bottled or mineral water were selected.

### Case study based on myfood24 validation cohort

#### Study sample and dietary recall data

The myfood24 validation cohort [10] (n = 212) was used to explore the GHG emissions associated with diet in a UK-based free-living adult population, using a database of ~40,000 food items. This validation study reported on results from an interviewer-based 24hr recall, and the online myfood24 24hr recall compared against reference measures from biomarkers [10]. Metabolically stable participants between 18 and 65y broadly representative of the UK population were recruited via the North West London Primary Care Research Network, posters in local doctors’ surgeries and postal addresses. Each 24hr recall and set of biomarker measurements were recorded on up to three different occasions separated by approximately two weeks.

#### Estimating diet-related GHG emissions

Participants recorded between one and three days of dietary intake. GHG emissions estimates accounted for quantities of food eaten by multiplying the GHG emissions estimate per kg food by the size of the portion reported, to give estimates in terms of absolute CO_2_ equivalents emitted per day (kg CO_2_eq / day). A mean GHG emissions value was calculated per 24hr recall completed by each person by summing the GHG emissions estimate across all foods eaten each day, averaged over the number of days recorded.

Foods were categorised by their dominant component, for example, sausages were grouped with meat, even if they contained a small proportion of cereal. Some composite dishes that could not be allocated to any other dominant category were categorised as “other”. Tea and coffee were categorised as drinks, but any added milk was categorised as dairy.

### Statistical analysis

To address skewness and non-constant variance, GHG emissions were log-transformed in all linear regression models, with estimates subsequently back-transformed and presented as percentage difference in geometric means. Comparisons of GHG emissions by food category were made by sex, age group, body mass index (BMI) group, and vegetarian status.

Intakes of selected nutrients were compared to World Health Organization (WHO) Recommended Nutrient Intakes (RNIs) [13, 14]. Diet-related GHG emissions for those not meeting recommended intakes were compared to those who were using linear regression models. Models were repeated both unadjusted for potential confounders and adjusted for age, sex and body mass index.

All analyses were conducted using Stata version 15 [15]. Statistical significance was set at p<0.05.

## Results

The database associated with the online dietary assessment tool myfood24 includes 3287 generic food items. GHG emissions values were assigned to 3233 (98%) items based on values reported in Poore & Nemecek [12] (n = 1981) and Murakami & Livingstone [4] (n = 1244), with additional values from Green et al. [6] (n = 5). The 54 (2%) foods missing a GHG emissions value were primarily stocks, sauces and dressings.

The cohort (n = 212) has been described and discussed elsewhere [10]. Briefly, 212 participants provided complete myfood24 online 24hr recalls and biomarker samples on at least one occasion. This comprised 128 (60%) participants with 3 days’ worth, 69 (33%) with 2 days’ worth, and 15 with 1 days’ worth of recalls. This may affect an individual’s chances of meeting the recommendations on that particular day (either increasing or decreasing them) but does not affect the analysis of the overall % meeting recommendations at the population level. Demographic characteristics of the cohort are shown in Table 1. The mean age was 43y, 127 (60%) were female, and 15 (7%) participants self-declared as being vegetarian. The participants collectively consumed 1313 different food items. Of these 1279 (97%) were assigned GHG emissions values.

**Table 1:** Demographic characteristics of participants by sex.

	Women	Men
	(n = 127)	(n = 85)
*Age (years)*		
<40	54 (43%)	33 (39%)
40+	73 (57%)	52 (61%)
*Body mass index (BMI)(kg/m* ^*2*^ *)*		
<25	72 (57%)	34 (40%)
25+	55 (43%)	51 (60%)
*Age left education (years)*		
16 or under	11 (9%)	13 (15%)
17 to 18	36 (28%)	23 (27%)
19+	78 (61%)	49 (58%)
*Ethnicity*		
White	92 (72%)	63 (74%)
Other	31 (28%)	21 (26%)
*Smoking status*		
Non-smoker	99 (78%)	65 (76%)
Smoker	13 (10%)	12 (14%)
*Vegetarian status*		
Yes	9 (7%)	6 (8%)
No	113 (93%)	72 (92%)
*Diet-related GHG emissions (kg CO*_*2*_ *eq/day)*		
5 or under	54 (43%)	22 (26%)
5–9	55 (43%)	34 (40%)
10+	18 (14%)	29 (34%)
*Arithmetic mean diet-related GHG emissions (kg CO*_*2*_ *eq/day)(95% CI)*	6·2 (5·6, 6·7)	9·3 (7·9, 10·7)
*Geometric mean diet-related GHG emissions (kg CO*_*2*_ *eq/day)(95% CI)*	5·4 (5·0, 5·9)	7·6 (6·7, 8·7)

Mean greenhouse gas emissions associated with a person’s daily diet were 7.4 kg CO_2_eq/day (95% CI 6.7 to 8.1), with a geometric mean of 6.2 kg CO_2_eq/day (95% CI 5.8 to 6.7). The largest overall contribution to diet-related GHG emissions came from meat (32% of total diet-related GHG emissions), with drinks (15%), dairy (14%), and cakes, biscuits & confectionery (8%) also contributing relatively high amounts. The GHG emissions from drinks were dominated by tea, coffee and alcoholic beverages which, together with cakes, biscuits and confectionary, suggest 24% of total diet-related GHG emissions derive from largely optional foods and drinks.

**Fig 1:**
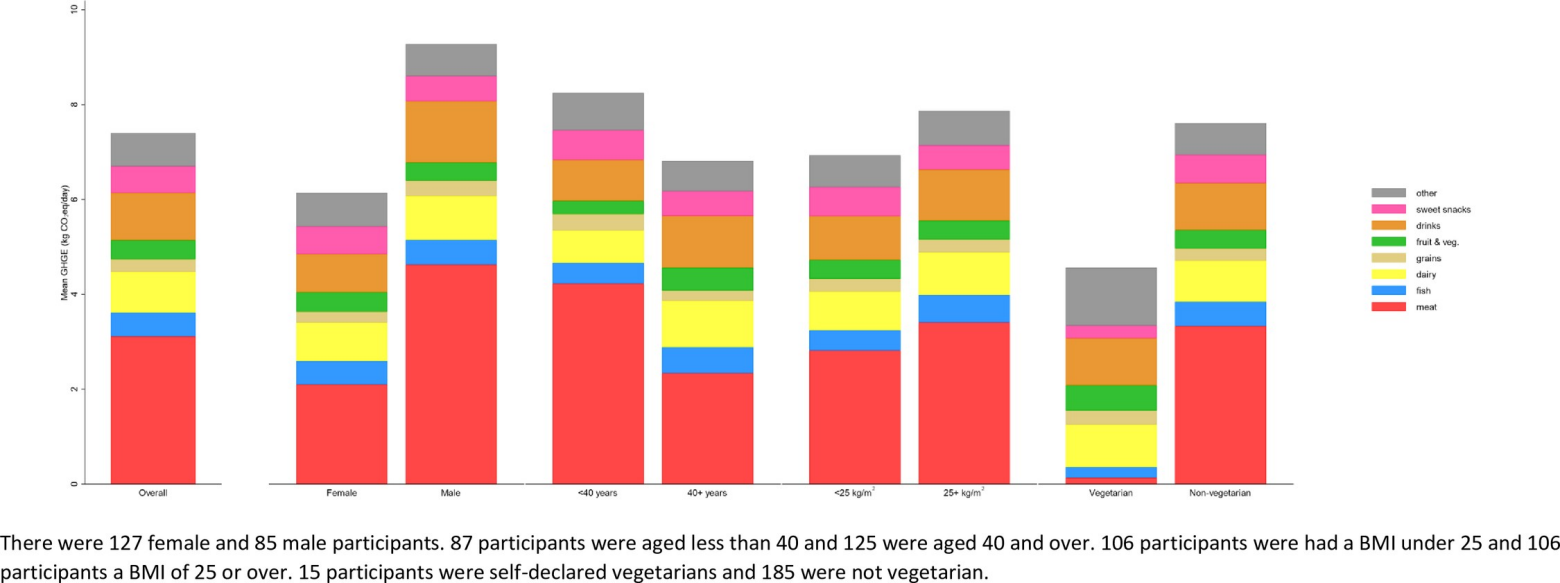
Contribution of different types of food to greenhouse gas emissions from diet by age, sex, body mass index and vegetarian status.

The diets of men were associated with 41% higher GHG emissions than women (95% CI 20% to 64%), driven by differences in meat intake and to a lesser extent by GHG emissions from drinks (Fig 1, S1 Table). There was no evidence of a difference between the GHG emissions from diets of people under 40 years and those 40 or over (mean difference 8%, 95% CI -8% to 27%), nor evidence of a difference between the GHG emissions from diets of people who were overweight or obese compared to those who were not (mean difference 13%, 95% CI -4% to 32%).

Non-vegetarian diets were associated with 59% higher GHG emissions than vegetarians (95% CI 18% to 115%), with the difference again driven by meat intake. Vegetarians also had lower GHG emissions associated with cakes, biscuits and confectionery, reflecting healthier dietary patterns more generally. Small increases in GHG emissions associated with fruit and vegetables and other foods amongst vegetarians did not detract from the lower contribution from meat and fish. There was no evidence of difference in dairy intake between vegetarians and non-vegetarians.

There was evidence that cohort participants exceeding the RNI for saturated fat had diets associated with higher GHG emissions (mean difference 22%, 95% CI 1% to 48%). Participants not achieving the RNI for carbohydrates had diets associated with higher GHG emissions (mean difference 56%, 95% CI 26% to 94%) after adjusting for age, sex and BMI. Participants who were exceeding the RNI for sodium had substantially higher diet-related GHG emissions (mean difference 74%, 95% CI 13% to 167%). Each of these associations is consistent with meat being the major contributor to the GHG emissions value. There was insufficient evidence of association with other RNIs (see Table 2).

**Table 2:** WHO recommended nutrient intakes for selected nutrients and comparison between those meeting and those not meeting these recommendations.

	Recommended Nutrient Intake (RNI)		Comparator Nutrient Intake		% difference in GHG emissions between RNI and comparator	
Nutrient	Recommended Intake (n)*	Geometric mean GHG emissions (kg CO_2_eq/day)	Comparator Intake (n)*	Geometric mean GHG emissions (kg CO_2_eq/day)	Unadjusted**	Adjusted***
		(95% CI)		(95% CI)		
Total fat	15–30% energy	6·0 (4·8, 7·4)	Over RNI	6·3 (5·8, 6·9)	6% (-14%, 30%)	7% (-12%, 31%)
	(n = 40)		(n = 172)			
Saturated fat	<10% energy	5·1 (4·3, 6·1)	Over RNI	6·6 (6·0, 7·2)	30% (8%, 57%)	22% (1%, 48%)
	(n = 47)		(n = 165)			
Protein	10–15% energy	5·8 (5·1, 6·6)	Over RNI	6·7 (6·0, 7·4)	15% (-3%, 35%)	8% (-8%, 28%)
	(n = 75)		(n = 129)			
Carbohydrates	55–75% energy	4·2 (3·3, 5·4)	Under RNI	6·6 (6·1, 7·2)	57% (26%, 95%)	56% (26%, 94%)
	(n = 30)		(n = 182)			
Fibre	25g minimum	6·9 (6·2, 7·7)	Under RNI	5·8 (5·2, 6·5)	-15% (-28%, -1%)	-10% (-23%, 6%)
	(n = 85)		(n = 127)			
Total sugars	Recommend <5% energy, Max. 10% energy	6·7 (5·0, 9·0)	Over maximum RNI	6·2 (5·7, 6·7)	-8% (-29%, 18%)	-2% (-24%, 26%)
	(n = 23)		(n = 189)			
Sodium	2000mg maximum	3·4 (1·6, 7·3)	Over RNI	6·4 (5·9, 6·9)	85% (20%, 187%)	74% (13%, 167%)
	(n = 7)		(n = 205)			

* Numbers (n) provided show the number meeting the RNI and the number not meeting the RNI (comparator group).

** % difference shows the difference between the comparator and those who meet the RNI. For example, in the case of total fat this is the % difference between those who are over the RNI for fat vs those who are meeting the RNI for total fat. For carbohydrates it is the % difference between those who are under the RNI vs those who are meeting the RNI.

*** Adjusted for age, sex, and body mass index.

## Discussion

Our study has identified and linked GHG emissions to 3233 generic food items from the national UK composition of foods integrated dataset [8]. In linking these to a fully automated online dietary assessment tool, we demonstrate the feasibility of deriving measures of both environmental sustainability and nutrient intake from detailed individual diets at scale. This has allowed us to quantify diet-related GHG emissions and compare different population subgroups. We found that nearly a quarter of GHG emissions were associated with elements of the diet that are nutritionally optional, such as drinks, cakes, biscuits and confectionery. We also confirmed the lower GHG emissions from vegetarian diets compared to omnivorous diets, which were largely attributable to differences in meat consumption. Our findings are consistent with previous reviews that found animal-based foods had greatest environmental impact [16], specifically beef [17].

Previous studies have based their results on a reduced set of food items [18, 19] or from broad groupings that combine foods with very different emission profiles [4]. This provides adequate coverage of population intakes, but imprecise application to quantifying GHG emissions for individuals. Such studies were able to show that beef, lamb and other red meats contribute significantly to GHG emissions [4, 18, 19]. We have extended this methodology substantially to over 3000 generic food items and 40000 branded items, allowing greater precision for estimating individuals’ environmental impact. Our findings confirm those based on broader food groupings in that red meat is a major driver of dietary-related GHG emissions. However, our study adds the potential for modelling subtle changes in dietary practice and minimise the risk of information loss caused by aggregating food types.

The 7.4 kg CO_2_eq/day mean daily GHG emissions value for a person’s daily diet derived from our sample was broadly consistent with the literature. In their cross-sectional study based on UK National Diet and Nutrition Survey data, Murakami & Livingstone [4] reported mean value of 8·2 kg CO_2_eq/day. We found no evidence of higher dairy intake in vegetarians, such as substitution of protein from meat with protein from cheese-based foods, which would have undone some of the gains from lower intakes of meat and fish. Similarly, Masset et al. [18] found no difference in dairy intake between ‘more sustainable’ and ‘average’ diets. Previous studies have suggested that higher emissions in men are attributable to a greater volume of food consumed [18], with greater overall energy intake [4]. However, in our analyses, higher diet-related GHG emissions in men were largely driven by higher absolute intakes of meat, not by other energy-rich foods such as fish, dairy, grains or sweet snacks.

We also show that diets meeting a range of RNIs generally have lower GHG emissions than those not meeting the RNIs. For example, diets meeting recommendations for lower saturated fat, lower sodium intake and a larger proportion of total energy intake from carbohydrates, were also lower in meat. Based on our results for the nutrients studied, future efforts to optimise diets for personal and planetary health may result in both nutritional and environmental benefits.

Several modelling studies conclude that healthy and sustainable diets can be jointly achieved through dietary optimisation [20–22]. Research shows that nutritionally optimised diets can have a lower environmental impact, but that this is not a linear relationship. Green et al. suggested that if average UK diets met WHO dietary guidelines, GHG emissions would reduce by 17%. However, evidence suggests that GHG emissions reductions above 40% would require substantial change and could compromise dietary quality [6]. More recently, Scheelbeek et al. showed that greater adherence to the UK Eatwell Guide was associated with both population health benefits and lower GHG emissions, but not with a reduced water footprint [23]. Reynolds et al. [24] found it theoretically possible to create a nutritionally optimised diet with a 57% GHG emissions reduction. However, to achieve the current UK government target of 80% reduction by 2050, major changes in both diet and food production would be needed. This pattern is evident in the global literature, which also shows that dietary shifts can reduce GHG emissions [25–27]. The literature discussed is useful but limited in the range of GHG emissions, generally aggregating foods to the subgroup level and using an average GHG emissions value. Similarly, individuals are grouped by sex [6] or by income [28] and Reynolds et al. [24] use household purchase data to derive food intake. Consequently, diets are not assessed in those studies on an individual level. A potential solution to drive GHG savings is a carbon tax on high GHE foods like red meat [29]. However, without a simple, evidence-based ‘climate smart’ diet and no means to accurately assess this on an individual intake level, the implementation of this remains problematic.

Whilst 24-hour recalls are an established means of measuring diet precisely over the days covered, allowing estimates of average intakes, there may be misclassification of individuals meeting the RNI on any one day. In addition, to accurately assign GHG emissions values to foods and subsequently calculate valid mean daily GHG emissions values for individual diets, further questions need to be asked related to dietary assessment. GHG emissions values can vary for the same food item depending on how and where it is produced; with the largest GHG emitters having up to 50 times greater emissions than the lowest [12]. Information relevant to the setting of GHG emissions values includes seasonality and country of origin, both of which impact on the emissions related to transport and the amount of energy used in the production of the food. At an even finer scale, the specific location of the farm influences its production efficiency due to differences in the local environment and microclimate, and different production methods (e.g., organic vs intensive conventional) affect the emissions. However, it is not always clear whether this information is available on a granular level, and it is therefore typically not possible to identify emissions per product other than at a broadly aggregate level.

At one end of the life cycle, food production method affects the environmental impact of a food; at the other end of the life cycle, purchase, transport to home and cooking method may influence a food’s footprint. Whilst the LCA data used in this study was very complete, extrapolations can affect the results due to data uncertainty. Our GHG emissions values included processing stages up to the point of retail, but not in the home. ‘In home’ data could be gathered at the dietary assessment stage; however, consumers are unlikely to be able to fully provide this information. Seasonality and purchase, transport to home, cooking method and information on food waste may be asked of participants directly, whereas country of origin and organic status could be taken from information included on product packaging. Production method may be harder to determine; this is something that policymakers could consider to make the environmental credentials of foods more transparent. Use of ‘blockchain’ technology is in its infancy but is increasingly being developed to track food production stages in a standardised way [30]. This is also relevant to the issue of transport; recent evidence suggests that shorter, regional supply chains are necessary to reduce environmental impact and limit global warming [31].

A strength of this research is the number of foods that have been assigned GHG emissions values, which goes beyond much of the current literature. The myfood24 database includes over 40,000 generic and branded food items. We have assigned GHG emissions values to the vast majority (98%, n = 3233) of the 3287 generic foods in this database, which were used to populate the remaining branded items using the closest approximating value. This gives great potential for future work to further use the branded product information to assign unique values, increasing the precision and accuracy of assessing the GHG emissions impact of individual diets. We add to the current literature, covering a large number of food items whilst moving beyond production stages of the life cycle, and reflecting both *what* was and the *amount* eaten, by individuals in their mean daily GHG emissions value. This generates GHG emissions values that relate more closely to individual diets and provide greater scope for future optimisation.

In this research, GHG emissions values assigned to generic food items included process stages up to the point of retail. This end point was chosen because it accounts for all food production stages outside the consumer’s control but does not include the transport to home or food waste stages associated with the individual. Although using a cradle to retail approach may underestimate the impact of total diet, the excluded stages are highly variable between individuals and are currently not sufficiently accounted for in dietary assessment to accurately quantify with a GHG emissions value. Excluding these stages prevents inaccuracies that could skew the overall environmental impact value of a particular food. Additionally, this cut-off point corresponded with the main source of GHG emissions values [12], which also used retail as the process stage cut-off. Within the cohort sample itself, there was a low number of self-declared vegetarians, which will create wide confidence intervals for this group.

The GHG emissions values assigned to food items in this research accounted for a comprehensive range of factors up to the point of retail, including production method, land use management, feed used, soil and climate, processing and transport of both the product and aspects of its production e.g., fertiliser and feed. However, GHG values were taken from multiple sources, and the consistency of impact estimates for each factor, such as transport, and the importance of this, is uncertain. In addition, these values are based on food items sold in the UK, so may not be accurate for the same foods sold in other countries. Furthermore, should trade patterns change in the wake of Brexit or the COVID-19 pandemic, the accuracy of these GHG emissions value estimates may change, as the algorithm behind the GHG estimates for foods using Poore & Nemecek [12] accounted for non-UK derived LCAs and was devised before these events.

Much of the literature is limited by a narrow Western Europe focus on a single environmental indicator such as GHG emissions, and by methodological differences between LCAs [12]. Our research also only considers GHG emissions, though in future research the algorithm in Poore & Nemecek [12] can be applied to other indicators such as land use, acidifying emissions, eutrophying emissions and water footprint. Although GHG emissions is where associations between health and environmental gains are strongest [3], multiple environmental impacts need consideration to ensure cohesion within the food production system. For example, although nuts and olive oil have a relatively low GHG emissions impact, water use is high [32]. Accounting for multiple indicators also allows engagement with various UN Sustainable Development Goals (SDGs). The SDGs cannot be met if, for example, SDG 2 (zero hunger) is pursued via production efficiency, as SDGs 15 (land), 6 (water), 13 (climate), 14 (sea) and 3 (health) would be compromised [33].

## Conclusion

This paper addresses the environmental impact of diet on the individual level, based on the GHG emissions value of foods consumed. It has tested and established the feasibility of populating a large online food and nutrient product database with GHG emissions values. Meat was the dominant driver for diet-related GHG emissions, explaining most of the differences between GHG emissions associated with vegetarian and non-vegetarian diets, and between the differences in GHG emissions associated with the diets of men and women. However, drinks such as tea and coffee, and cakes, biscuits and confectionery, explained a quarter of diet-related GHG emissions, and present alternative routes to reduce diet-related GHG emissions. Those who met dietary recommendations had generally lower diet-related GHG emissions, suggesting future policies to encourage sustainable dietary patterns and plant-based diets could be good for both individual and planetary health.

## Supporting information

S1 Table Mean (95% CI) and percent contributions of different types of food to greenhouse gas emissions from diet by age, gender, body mass index and vegetarian status (kg CO2eq/day). (PDF)

S1 Appendix Summary of key references containing greenhouse gas (GHG) emissions values. (XLSX)

S2 Appendix References listed in key papers used to derive greenhouse gas (GHG) emissions values. (XLSX)
