# Supplementary material for: Variations in greenhouse gas emissions of individual diets: Associations between the greenhouse gas emissions and nutrient intake in the United Kingdom
Source: PLoS One. 2021 Nov 23;16(11):e0259418. doi: 10.1371/journal.pone.0259418 (PMC8610494; doi:10.1371/journal.pone.0259418)
Supplement: S1 Table — (PDF) [file pone.0259418.s001.pdf]

Supplementary table 1· Mean (95% CI) and percent contributions of different types of food to greenhouse gas emissions from diet by age, gender, body mass index and vegetarian status (kg CO_2eq/day)

	Total	Sex		Age		BMI		Vegetarian status	
		Female	Male	<40 years	40+ years	<25 kg/m^2	25+ kg/m^2	Vegetarian	Non-vegetarian
**Meat**	3·1 (2·5, 3·7) 32%	2·1 (1·6, 2·6) 27%	4·6 (3·3, 6·0) 38%	4·2 (2·9, 5·5) 37%	2·3 (1·8, 2·9) 27%	2·8 (1·9, 3·7) 29%	3·4 (2·5, 4·3) 34%	0·1 (0·0, 0·3) 2%	3·3 (2·6, 4·0) 34%
**Fish**	0·5 (0·4, 0·6) 7%	0·5 (0·3, 0·6) 8%	0·5 (0·3, 0·7) 6%	0·4 (0·3, 0·6) 6%	0·5 (0·4, 0·7) 8%	0·4 (0·3, 0·6) 7%	0·6 (0·4, 0·8) 8%	0·2 (0·0, 0·5) 3%	0·5 (0·4, 0·6) 7%
**Dairy**	0·9 (0·8, 0·9) 14%	0·8 (0·7, 0·9) 15%	0·9 (0·8, 1·1) 13%	0·7 (0·6, 0·8) 12%	1·0 (0·8, 1·1) 15%	0·8 (0·7, 0·9) 15%	0·9 (0·8, 1·0) 14%	0·9 (0·6, 1·2) 22%	0·9 (0·8, 1·0) 14%
**Grains**	0·3 (0·3, 0·5) 4%	0·2 (0·2, 0·3) 4%	0·3 (0·2, 0·4) 4%	0·3 (0·3, 0·4) 5%	0·2 (0·2, 0·3) 4%	0·3 (0·2, 0·3) 4%	0·3 (0·2, 0·3) 4%	0·3 (0·1, 0·4) 7%	0·3 (0·2, 0·3) 4%
**Fruit and vegetables**	0·4 (0·3, 0·5) 7%	0·4 (0·3, 0·5) 7%	0·4 (0·3, 0·5) 6%	0·3 (0·2, 0·3) 5%	0·5 (0·4, 0·6) 8%	0·4 (0·3, 0·5) 7%	0·4 (0·3, 0·5) 6%	0·5 (0·3, 0·8) 13%	0·4 (0·3, 0·5) 6%
**Drinks**	1·0 (0·9, 0·7) 15%	0·8 (0·7, 0·9) 15%	1·3 (1·0, 1·5) 16%	0·9 (0·7, 1·0) 13%	1·1 (0·9, 1·3) 17%	0·9 (0·7, 1·1) 15%	1·1 (0·9, 1·2) 15%	1·0 (0·5, 1·5) 21%	1·0 (0·9, 1·1) 15%
**Sweet snacks**	0·6 (0·5, 0·7) 8%	0·6 (0·4, 0·7) 9%	0·5 (0·4, 0·7) 7%	0·6 (0·4, 0·8) 9%	0·5 (0·4, 0·6) 8%	0·6 (0·4, 0·8) 10%	0·5 (0·4, 0·6) 7%	0·3 (0·1, 0·4) 6%	0·6 (0·5, 0·7) 9%
**Other**	0·7 (0·6, 0·8) 11%	0·7 (0·5, 0·9) 12%	0·7 (0·5, 0·8) 10%	0·8 (0·5, 1·0) 13%	0·6 (0·5, 0·8) 11%	0·7 (0·6, 0·8) 12%	0·7 (0·5, 0·9) 11%	1·2 (0·1, 2·4) 24%	0·7 (0·6, 0·8) 11%
